# Applying Factor Analysis Combined with Kriging and Information Entropy Theory for Mapping and Evaluating the Stability of Groundwater Quality Variation in Taiwan

**DOI:** 10.3390/ijerph8041084

**Published:** 2011-04-08

**Authors:** Guey-Shin Shyu, Bai-You Cheng, Chi-Ting Chiang, Pei-Hsuan Yao, Tsun-Kuo Chang

**Affiliations:** 1 Department of Environmental Management, Tungnan University, Taipei County 222, Taiwan; E-Mail: gsshyu@mail.tnu.edu.tw; 2 Ecological Engineering Research Center, National Taiwan University, Taipei City 106, Taiwan; 3 Department of Bioenvironmental Systems Engineering, National Taiwan University, Taipei City 106, Taiwan; E-Mails: f92622003@ntu.edu.tw (C.-T.C.); d97622006@ntu.edu.tw (P.-H.Y); tknchang@ntu.edu.tw (T.-K.C.)

**Keywords:** Kriging, factor analysis, information entropy theory, groundwater, GIS

## Abstract

In Taiwan many factors, whether geological parent materials, human activities, and climate change, can affect the groundwater quality and its stability. This work combines factor analysis and kriging with information entropy theory to interpret the stability of groundwater quality variation in Taiwan between 2005 and 2007. Groundwater quality demonstrated apparent differences between the northern and southern areas of Taiwan when divided by the Wu River. Approximately 52% of the monitoring wells in southern Taiwan suffered from progressing seawater intrusion, causing unstable groundwater quality. Industrial and livestock wastewaters also polluted 59.6% of the monitoring wells, resulting in elevated EC and TOC concentrations in the groundwater. In northern Taiwan, domestic wastewaters polluted city groundwater, resulting in higher NH_3_-N concentration and groundwater quality instability was apparent among 10.3% of the monitoring wells. The method proposed in this study for analyzing groundwater quality inspects common stability factors, identifies potential areas influenced by common factors, and assists in elevating and reinforcing information in support of an overall groundwater management strategy.

## 1. Introduction

Several water quality items define water quality characteristics. Several researchers have undertaken multivariate analyses to understand hydro-geological characteristics and contamination of regional groundwater [1–7]. Factor analysis extracts the multivariate influence to understand the cause(s) of major factors affecting water quality, and to acquire information on the strength of the influence. Researchers have recently adopted spatial technology as an important analytical tool to describe and map the spatial variability of hydro-chemical parameters [4,8–11]. In collecting data, each spatial sampling position S(*x*, *y*) may be used to measure the multivariate factors (*v*_1_, *v*_2_, *v*_3_, …, *v*_n_), and each variable may contain multiple sampling records on different frequencies (*v*_11_, *v*_12_, *v*_13_, …, *v*_nt_), where *t* is frequency. The analysis unit for considering the characteristics of water quality and spatial correlations should be based on one measurement such as data from a single survey, the average, or the median value during a long-term investigation. Many studies have emphasized spatial analyses of water quality data, but ignored temporal information [8,9,12–14]. Therefore, this study includes information stability for each monitoring well will to understand the spatial variation of well stability.

The characteristics of groundwater quality closely relate to environmental variability. Cruz and Silva analyzed the groundwater data set for Pico Island (Portugal) inferring that silicate mineral dissolution and water salinization were mainly responsible for observed changes in groundwater composition [15]. Aiuppa *et al.* analyzed groundwater from Mr. Etna, Italy and revealed three major sources of groundwater contaminants: leachate from the host basalt, saline brines from the sedimentary basement below Mt. Etna, and agricultural and municipal wastewaters [12]. Kim *et al.* used the modified piper diagram to investigate salinization of shallow groundwater in the coastal reclaimed regions of Korea and reported that residual salts from seawater intrusion, and organic matter in the filling materials accelerated the groundwater salinization process [16].

Studies using factor analysis to assess groundwater quality have shown that extracted factors are often related to the saline parameters of groundwater quality. Adams *et al.* used factor analysis to assess groundwater in the Western Karoo (South Africa) and its interaction with the environment, and reported that the salinization process, mineral precipitation and dissolution, cation exchange, and human activity were the main processes influencing groundwater quality [17]. Kim *et al.* divided shallow groundwater in the coastal area at Kimje City (Korea) into four groups and revealed that seawater intrusion, chemical fertilizers, and the reduction process affected physicochemical compositions of groundwater [18]. Liu *et al.* investigated groundwater quality in the coastal Blackfoot Disease (BFD) area in Yun-Lin County (Taiwan) and discovered that groundwater quality was mainly controlled by seawater intrusion and arsenic pollution [9]. Additionally, the areas of high salinization and arsenic pollution were consistent with the area of groundwater over-pumping. Liu *et al.* demonstrated that brine groundwater was primarily composed of highly evaporated seawater [8]. However, the salinization factor did not determine the analysis results of groundwater samples in the contaminated sites. Subbarao *et al.* analyzed the effluent contamination of groundwater around a zinc (Zn) smelter plant and a polymer plant at Visakhapatnam (India), showing that the groundwater at the Zn smelter plant was contaminated by magnesium (Mg) and sulfate (SO_4_), whereas sodium (Na), chloride (Cl), and carbonates (CO_3_) were the major elements transported into groundwater at the polymer plant [14]. Love *et al.* applied factor analysis to prove that groundwater quality around an iron (Fe) mine and municipal sewage disposal plant in Southern Africa was related to agricultural activities, mining activities, and chemical usage [13].

Studies often combine factor analysis with cluster analysis for conducting spatial variance analysis. Cluster analysis is used to split water samples into a number of groups according to similar hydro-geochemical composition [2,18–20]. A large-area research typically integrates cluster analysis with geographical information system (GIS) technology to investigate whether the cluster phenomenon exists spatially. If so, factor analysis is then used to discuss the factor influence in each cluster to solve the less prominent factor influence of spatial variances in small areas [4,8,18,21–23].

The reported concentration values used for carrying out the multivariate statistical approach based on a single sampling or the statistics at each monitoring well do not include information on raw data uncertainty. The general results only display the groundwater quality characteristics for the specific time of the survey. Because groundwater quality may vary over time, the above results thus do not represent a realistic groundwater quality state. The ignored information may not change the factor component composition, but it will provide other useful information [8,9,13]. Therefore, this study includes factor influence. Shannon proposed entropy as a measure of uncertainly, a theory recently applied in various fields [24]. Although research has quantified the entropy theory to evaluate uncertainty for hydrological variables and parameters in models of water resources systems [25], studies have not fully explored its application for describing and evaluating large-scale characteristics of groundwater quality. Entropy theory establishes and quantifies uncertainty information to solve water resource and environmental management problems [26].

The three objectives of this study included: (1) applying cluster analysis and GIS technology to evaluate whether a spatial cluster phenomenon exists; (2) using factor analysis combined with kriging to interpret and map major factors that affect groundwater quality in Taiwan, and (3) investigating the stability and spatial variation of influential factors.

## 2. Materials and Methods

### 2.1. Study Area

Taiwan is approximately 36,000 square kilometers is extension, with 32% of the whole island having mean sea level elevations higher than 1,000 meters. The average annual precipitation is about 2,150 mm; the majority of this precipitation occurs from typhoons during the wet season from May to October. However, the spatial and temporal distributions of precipitation are extremely uneven, leading to a great difference in river flow during the dry and wet season. In Taiwan, the groundwater aquifer belongs mostly to quaternary sediments. Geologically, the groundwater aquifer of Taiwan is made up of coastal terrace, river terrace, and alluvial plain; the plain areas are mostly alluvial fans with abundant groundwater. The distribution of groundwater resources in Taiwan are divided into nine groundwater areas. The water resources come from surface water (68%) and groundwater (32%) to supply 70% of agricultural, 21% of domestic, and 9% of industrial water demands [27]. The stable quantity of groundwater deems it an important water resource.

### 2.2. Hydro-Geochemical Dataset

The regional and site-specific groundwater monitoring wells established by the Taiwan Environmental Protection Administration (EPA) and the monitoring wells established by the Water Resources Agency (WRA) constitute the main groundwater monitoring networks in Taiwan. Since 1999, the Taiwan EPA has continually established regional monitoring wells (depth less than 20 meters) with a density of 0.4 wells/100 hectares for seasonal sampling. These wells are mainly used as a pre-warning system for groundwater contamination. The procedure for this study purged groundwater with three times the volume of wells to remove suspended solids before collecting groundwater samples. Samples were collected with bailers, stored in polyethylene bottles, and preserved according to standard analytical methods (EPA). The sample bottles were placed in 4 °C containers and transported to the laboratory for analysis. Some relatively unstable hydrochemical parameters such as temperature (Temp.), pH and electrical conductivity (EC) were measured in the field. The general hydrochemical parameters included: total hardness (TH), total dissolved solid (TDS), chloride (Cl^−^), ammonia (NH_3_-N), nitrate (NO_3_-N), sulfate (SO_4_^2−^) and total organic carbon (TOC). The metal parameters included arsenic (As), cadmium (Cd), chromium (Cr), copper, (Cu), lead (Pb), zinc (Zn), iron (Fe), and manganese (Mn). Since 2005, the supplemental five parameters have included calcium (Ca^2+^), magnesium (Mg^2+^), sodium (Na^+^), potassium (K^+^), and alkalinity (Alk.), for a total of 23 analytical parameters.

**Figure 1.**
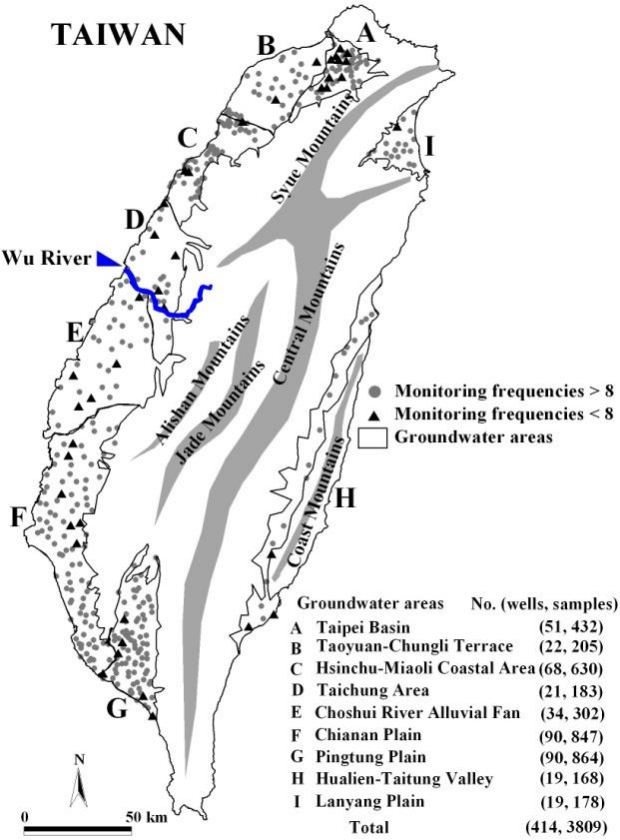
Location of monitoring wells in groundwater areas.

This study analyzed groundwater samples collected seasonally from 2005–2007, derived from 414 regional monitoring wells shown in Figure 1. In general, each monitoring well was sampled and analyzed quarterly. Sometimes samples could not be collected due to natural or human interruption causes. As a result, this study collected eight or more samples from about 90% of the monitoring wells (373) to provide stable and effective hydrochemical data.

### 2.3. Statistical and Geostatistical Analysis

Cluster analysis selected two southern and northern areas for studying spatial evaluations. Factor analysis was applied to discover the factors influencing groundwater quality in these two areas. Then, entropy theory was used to quantitatively evaluate the stability of groundwater quality.

#### 2.3.1. Cluster Analysis (CA)

Cluster analysis is an unsupervised pattern detection method that classifies all cases into smaller groups or clusters based on similarities within a group and dissimilarities among different groups. Therefore, the magnitude of association is strong (homogeneity) between cases in the same group and weak (heterogeneity) among different groups. The similarity between two cases is typically quantified through Euclidean distance measurements. Hierarchical agglomerative clustering, which is the most common method, has the advantage of not making any prior assumptions about the data. The visual compendium of the clustering processes is typically displayed as a dendrogram (tree diagram). However, the fact that the user must decide the number of groups causes subjective judgments in the cluster analysis. This study employed hierarchical agglomerative cluster analysis on standardized data using Ward’s method with squared Euclidean distance. Ward’s method attempts to minimize the sum of squared distances of centroids from any two hypothetical groups formed at each step. The linkage distance is expressed as (D*_link_*/D*_max_*) × 100, which represents the standardized quotient between the linkage distances for a particular case divided by maximal linkage distance [11,28,29].

#### 2.3.2. Ordinary Kriging (OK)

Kriging is a group of geostatistical techniques used to interpolate the value of a random field at an unobserved location from observations of its value at nearby locations. The main tool of most geostatistical analyses is the variogram. The variogram can be defined as half the expected squared difference between paired random functions separated by the distance and direction vector. The important characteristics of the variogram are range, sill, and nugget effect. The variogram function can be expressed as follows:

(1) $$\hat{\gamma }(h,\;x)=\frac{1}{2m(h)}\;\sum_{i=1}^{m(h)}\left[\text{Z}(x_{i})-\text{Z}(x_{i}+h)\right]$$

where *m*(*h*) is number of pairs observations, Z(*x_i_*) represents the regionalized variable at position *x_i_*. For the traditional variogram, which is a function of one variable *h*, the model for the variogram can be obtained by the use of mathematical models for instance exponential, spherical, Gaussian, and linear variogram. These models may be fitted to the variogram and the coefficients of the model may be used to assign optimal weights for interpolation using kriging. In this study used form of kriging is ordinary kriging. Ordinary kriging assumes an unknown constant trend: μ(x) = μ. The estimate method is linear weighted moving averages of the n available observations. Then the interpolation by ordinary kriging is given by:

(2) $$\hat{\text{Z}}(x_{0})=\sum_{\text{i}=1}^{n}\lambda_{i}\;\text{Z}(x_{i})$$

(3) $$\sum_{\text{i}=1}^{n}\lambda_{\text{i}}=1$$

where Ẑ(*x_0_*) is estimated the value at *x_0_*. And the kriging weights of ordinary kriging fulfill the unbiasedness condition. The weighting factors can be determined by solving a non-linear optimization problem involving the minimization of the estimated error to the constraint by using the Lagrange multiplier. The variance of estimation error is defined by equation (5):

(4) $$\text{L}(\lambda_{\text{i}},\;\mu )=\text{Var}\;\left[\sum_{\text{i}=1}^{\text{n}}\lambda_{i}\;\text{Z}(x_{i})-\text{Z}({\text{x}}_{0})\right]-2\mu \left(\sum_{\text{i}=1}^{\text{n}}\lambda_{i}-1\right)$$

(5) $${\hat{\sigma }}_{O\;K}^{2}=\sum_{i=1}^{n}\lambda_{i}\;\gamma_{0i}+\mu$$

More detailed discussions and mathematical inference are provided by Journel and Huijbregts and Isaaks and Srivastava [30,31].

#### 2.3.3. Factor Analysis (FA)

Factor analysis is an extensively used multivariate statistical method to rearrange original variables into fewer underlying factors (also called common factors) to retain as much information contained in the original variables as possible. Unlike original variables factors are completely uncorrelated with each other. Hence, substituting these factors for the original variables can effectively reduce the overall complexity of large data. The eigenvalue quantifies the contribution of a factor to total variance. Factors are produced according to an eigenvalue analysis of the correlation matrix, and factor loadings and factor scores are the main measurements of factor analysis. The first step of factor analysis is to standardize the raw data and compute a correlation matrix of the variables from the standardized variables. The second step is to estimate the factor loadings that express the degree of closeness between the factor and variables. Factor loadings range from −1 to +1, with a larger absolute value indicating a stronger relationship between the respective factor and variable. Furthermore, Liu *et al.* proposed that classifying the factor loadings as strong, moderate, and weak corresponds to absolute loading values in the range of >0.75, 0.75–0.50 and 0.50–0.30 [9]. The last step linearly transforms factors associated with the initial set of loadings by factor rotation to maximize variable variances and to obtain a better interpretable loading pattern. Factor scores are computed for each individual case to represent the contribution of each factor in each case.

This study performed factor analysis to determine the factors controlling regional groundwater composition of the two areas and the resulting factors in the main groundwater types. Factor extraction was carried out by principal components, where only eigenvalues greater than one were retained [32]. The factor loading matrix was rotated to obtain uncorrelated factors by varimax rotation.

This study considered factor scores of common factors in each groundwater monitoring well as variables and applied them to kriging methods to create various surfaces to display the range and degree of groundwater quality influenced by common factors.

#### 2.3.4. Information Entropy Theory

Shannon introduced the entropy concept into information theory by suggesting entropy as a measure of information or uncertainty. Shannon entropy expresses the degree of uncertainty implicated in predicting the output of a probabilistic event. Mathematically, an inverse relationship exists between the amount of information and the probability of occurrence. If the occurrence of an event can be precisely predicted the probability value will be great, and inversely, the Shannon entropy will be small. Hence, information and uncertainty as dual terms that reveal the information gained is indirectly measured as the amount of reduced uncertainty. Various fields of ecology, hydrology, and water quality have recently applied entropy theory [33–35].

The Shannon entropy can be explained as follows: let a set of n possible outcomes be *X* ∈{*x*_1_, *x*_2_,..., *x_n_*}, and the probabilities of the outcomes as *p*(*x*_1_), *p*(*x*_2_ ),..., *p*(*x_n_*) . The basic assumption of entropy is the amount of information, *H*(*X*), being a real non-negative measure, additive, and a continuous function of probability *p*. Therefore, entropy *H*(*X*) is defined as:

(6) $$H(X)=-\sum_{i=1}^{n}p_{i}\cdot \text{log}\;p_{i}$$

where *p_i_* is the probability of the outcome *x_i_*. In this study, the base of the logarithm was 2. The unit of entropy measurement is called a “bit.”

## 3. Results and Discussion

### 3.1. Descriptive Statistics

Table 1 shows the summary of statistics for the hydrochemical data in Taiwan. The water temperature ranged from 18.2 to 32.4 °C, with a maximum difference of 4 °C between the northern and the southern areas. The pH value of groundwater ranges from 3.6 to 10.5—the southern area groundwater is on the alkaline side of neutral, whereas the northern area groundwater is weakly acidic. The mean and standard deviation of other hydrochemical parameters are EC 2,366.4 ± 7,884.2 μS cm^−1^, TH 477.7 ± 1,017.6 mg L^−1^, and TDS 1,659.2 ± 5,832.9 mg L^−1^.

Table 1 shows the concentrations of Cd, Cr, Cu, and Pb in more than 80% of the samples, and Zn in 22% of samples were below the detection limits. More than 50% of shallow groundwater aquifers, equivalent to 60,000 hectares in the sampling area, contain As, indicating that groundwater in Taiwan generally contains trace amounts of As. Researchers have reported groundwater with high-arsenic concentrations in the southwestern coast of Taiwan, with the arsenic content of well water ranging from 0.01 to 1.82 mgL^−1^ High-arsenic concentrations are also in Blackfoot Disease hyperendemic areas [36–39]. The results in this study showed that the groundwater As concentrations ranged from N.D. to 0.191 mgL^−1^, where the highest concentration of As also occurred in Southwestern Taiwan.

Table 2 gives the correlation coefficient matrix for the hydrochemical parameters. If the correlation coefficient (*r*) is greater than 0.7, two parameters are considered to be strongly correlated, whereas if the *r* value is between 0.5 and 0.7, it indicates a moderate correlation at a significance level p < 0.05 [17]. Parameters having high degrees of correlations are EC and TDS (*r* = 0.998) because all of the dissolved components cause increased ionic concentration, as well as increased EC concentration. EC is highly related to TH (*r* = 0.970), Cl^−^ (*r* = 0.998), SO_4_^2−^ (*r* = 0.964), Na^+^ (*r* = 0.997), K^+^ (*r* = 0.996), and Mg^2+^ (*r* = 0.965) but moderately related to Ca^2+^ (*r* = 0.670).

The results indicated that these ions involve various physical and chemical reactions: e.g., oxidation/reduction reactions, and ion exchange in groundwater aquifers, which suggest that the same factor strongly affect them [40]. Ca^2+^ and SO_4_^2−^ have a relatively high correlation (*r* = 0.714), revealing that the calcium ion in groundwater comes mainly from gypsum. Na^+^ and Cl^−^ also have high correlation (*r* = 0.996). However, heavy metals having no significant correlations with other parameters are As, Cd, Cr, Cu, Pb, and Zn; therefore, As, Cd, Cr, Cu, Pb, and Zn are not included in the subsequent multivariate analyses. Only 17 water quality parameters (Temp., pH, EC, TH, TDS, Cl^−^, NH3-N, SO_4_^2−^, TOC, Fe, Mn, Na, K, Ca, Mg, and Alk) for samples collected from the 414 monitoring wells are used. Values for constituents lower than the methods detection limit (MDL) were replaced with half of the method detection limit (MDL/2) prior to statistical analysis to make up all data [41]. This study used the SPSS 13.0 software for multivariate statistical analysis and the results were plotted by ArcGIS 9.3.

### 3.2. Cluster Analysis of Spatial Correlation

**Figure 2.**
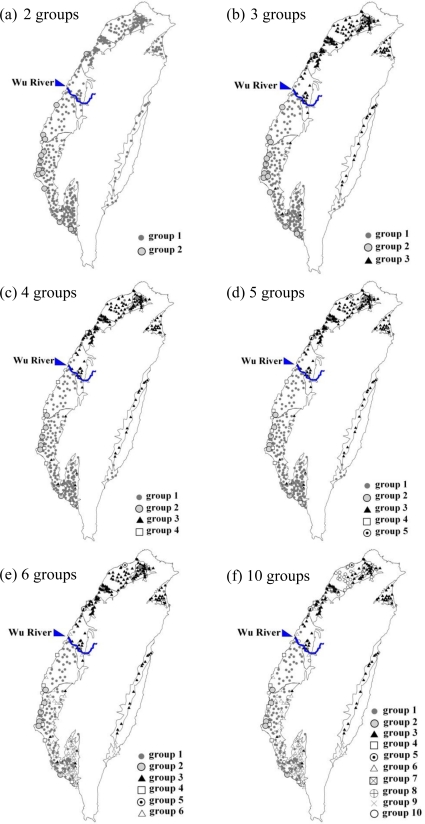
Overlay map of the spatial distribution of cluster analysis results.

The geological factor is the main factor affecting the groundwater by many researches [2,4–6,8,9,11,13,14]. In Taiwan, groundwater monitoring wells are distributed over nine groundwater aquifers with the farthest distance of 354 kilometers between two wells. This highlights the regional pattern of groundwater factors, which must reduce the global influence of common factors. However, common factors in the small area are appropriate to represent the real situation. Thus, cluster analysis and GIS technology were combined to determine if a spatial cluster exists. Based on the raw data, cluster analysis was performed to split the groundwater monitoring wells into two groups, three groups, and four groups up to multiple groups, and then plotted to determine the spatial distribution, shown in Figure 2.

When the monitoring wells were divided into two groups, the percentages of monitoring wells in the groups were 96% and 4%. Monitoring wells of the smaller group located in Southwestern Taiwan had the spatial aggregation as shown in Figure 2(a). When divided into three groups [ (D_link_ / D_max_ ) × 100 <10, D_link_ / D_max_ represents the quotient between the linkage distances for a particular case divided by the maximal linkage distance], the percentages of monitoring wells in each group were 56%, 40% and 4%. Figure 2(b) shows the first group aggregated south of the Choshui River Alluvial Fan, the second group in the Southwestern Taiwan, and the third group in the northern Taichung groundwater area and the eastern groundwater area. The three groups demonstrate distinct distributions in space. When the monitoring wells were divided into four and five groups, the spatial patterns were similar to those for the three previous groups, and only a few monitoring wells were divided into other groups [Figure 2(c) and 2(d)]. However, regardless of how the group increased the number of groups, as shown in Figure 2(e) and Figure 2(f), the division of groundwater characteristics into north and south groups is obvious. Therefore, in order to focus, choose to use the most appropriate area to be discussed.

Wells of the first group, identified as the northern area, were located in the Taipei Basin, the Taoyuan-Chungli Terrace, the Hsinchu-Miaoli Coastal Area, the Taichung Area, the Lanyang Plain, and the Hualien-Taitung Valley. Wells of the second group, the southern area, were located in the Choshui River Alluvial Fan, the Chianan Plain, and the Pingtung Plain. The northern area has 200 monitoring wells whereas the southern area has 214 monitoring wells. Because of the extreme value of the variation in groundwater quality, we compare the relative variation of both the northern and southern areas using a box-and whisker plot.

**Figure 3.**
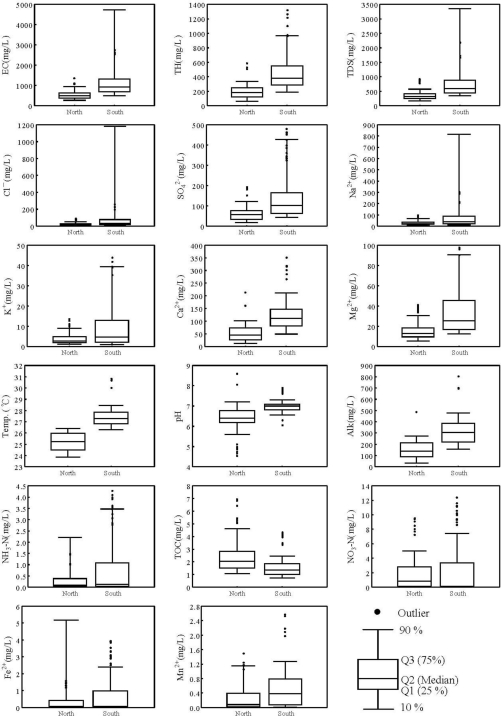
The box-and-whisker plot of various hydrochemical parameters for the northern and southern areas in Taiwan.

Figure 3 shows the box-and-whisker plot of hydrochemical parameters for the northern and southern areas. Based on the median value, the northern area had a greater concentration of TOC and NO_3_-N than the southern area, whereas the southern area had higher concentration of EC, TH, TDS, Cl^−^, SO_4_^2−^, Na^+^, K^+^, Ca^2+^, Mg^2+^, Temp., pH, Alk, Mn^2+^, NH_3_-N, and Fe^2+^ than the northern area. This demonstrated that factors affecting groundwater quality are different for the northern and the southern areas.

### 3.3. Factor Analysis Combined with Kriging

Factor analysis was performed on the normalized data sets (23 variables) separately for the northern and southern areas of Taiwan. This study retained only factors with eigenvalues that exceeded 1.0, and based on the absolute factor loadings, were greater than 0.625 to determine predominant parameters of the common factors. Table 3 presents the rotated common factors for the percentage of variance and the total cumulative percentage of variance.

#### 

##### Factor 1

For the northern area, Factor 1 consists of eight parameters: EC, TH, TDS, Cl^−^, SO_4_^2−^, Na^+^, K^+^, and Mg^2+^. For the southern area, Ca^2+^ is added to consist of nine parameters. Factor 1 explained 42.28% of the total variance for the southern area and 50.34% for the northern area. These parameters are the major ions in aqueous solution. Since EC can reflect the degree of groundwater pollution by seawater intrusion, we can regard it as a water salinization index [9]. The southern area suffered from discharge of agricultural and industrial wastewaters to groundwater and had higher groundwater EC value than the northern area, where groundwater in the southern area was already polluted.

##### Factor 2

Factor 2 accounts for 15.13% of total variance including the parameters pH, Ca^2+^, and Alk in the northern area. In the southern area, the association of TOC and Alk characterized factor 2 accounting for 12.19% of the total variance. Since the geology of the northern area is primarily limestone, the Ca^2+^ ions release into the groundwater, which changes the pH. The groundwater in the southern area has a wide distribution of TOC, mainly from livestock and industrial wastewater discharge. TOC is also a pre-warning of groundwater pollution. The source of alkalinity is different for the northern and the southern areas. In the northern area, Ca^2+^ ions are the main source of alkalinity. However, TOC degradation into inorganic carbon mainly causes alkalinity in the southern area.

##### Factor 3

Factor 3 for the northern area includes the parameters Fe^2+^ and Mn^2+^ explains 12.21% of the total variance. Factor 3 for the southern area includes only one parameter, Fe^2+^, which explains 8.44% of the total variance. The soil and rock in the groundwater are composed of Fe^2+^ and Mn^2+^. The iron dissolves in water to form divalent and trivalent iron cations and in the absence of other ions, the neutral and oxidizing water can form a ferric hydroxide deposit with the iron. Since the groundwater contains low amounts of dissolved oxygen, the anaerobic condition results in reduced trivalent iron and increased divalent iron in water. Hence, the groundwater contains higher concentrations of iron ions than surface water. Similarly, divalent manganese [Mn(II)] is the main type existing in groundwater.

##### Factor 4

Factor 4 in the northern area accounts for 10.80% of total variance, containing two parameters of NH_3_-N and TOC. The total organic carbon is a composite index that responds to the total mass of organic matter existing in water. In the southern area, Factor 4 only contains one parameter, temperature, which explains 7.76% of total variance. The groundwater contaminated by ammonia nitrogen results from organic matter contained in discharges of industrial, agricultural and domestic wastewaters decomposing into ammonia nitrogen by microbial reactions. The high concentration of ammonia nitrogen or organic nitrogen in water indicates water pollution. Inorganic ammonia is the main parameter of groundwater monitoring work and the existence of nitrogen compounds closely relate to organic matter. Ammonia nitrogen converts into nitrogen gas for release to the atmosphere through nitrification and denitrification, where nitrification is the key to the nitrogen cycle. Groundwater in the anaerobic condition precedes the nitrification process, leading to accumulated ammonia nitrogen in groundwater. Carbon and nitrogen originate from the same source, causing a high concentration of total organic carbon in groundwater.

The factor scores were evaluated using kriging method. Table 4 lists the variography results for common factor in northern area and southern area. A best-fit models with the lowest reduced sum of squares (RSS) and the highest R^2^ values were generated using GS+ software in order to fit variograms based on use of a least squares model. The varograms of F1, F2 in the northern area and F2, F4 in the southern area with high nugget effect ratios (>38.0%) represent high levels of small-scale variations. These variations may be due to the extreme observations in the study area. The spatial structures of northern area and southern area in common factors are not all similar.

### 3.4. Shannon Entropy Calculations

The current study analyzed the stability of each parameter using the information entropy theory to extend information contained in the long-term monitoring data. Some missing data for a few groundwater monitoring wells raised the data reliability by selecting monitoring wells with a sum greater than eight for data. The northern area has 175 groundwater monitoring wells, and the southern area has 198; a total of 373 groundwater monitoring wells occupied 90% of the whole data. This study calculated the information entropy for each selected groundwater monitoring well and ranked the groundwater monitoring wells according to their calculated information entropy values. Then the rankings of groundwater monitoring wells were summed up for each parameter classified by each common factor. Caused by the characteristics of different groundwater quality parameters, it is to rank to be added to replace the sum of entropy value. Table 5 shows the contained parameters. Finally, the magnitude of the sum of ranks was used to determine the stability of groundwater quality. The smaller value indicates a more unstable groundwater quality. Water quality variation in many groundwater-monitoring wells is not obvious, so these wells have the same rank. The groundwater parameters for these wells are relatively stable, and are not shown in the plot.

### 3.5. Evaluating Stability of Groundwater Quality Variation

Factor analysis of the scores can use the kriging method to draw contour maps. Factor scores can quantify common factors’ influence. A high score means the common factor has high-impact. With a well knowing the factor score and overlaying entropy, we can visualize the factors of influence and stability of the relationship.

**Figure 4.**
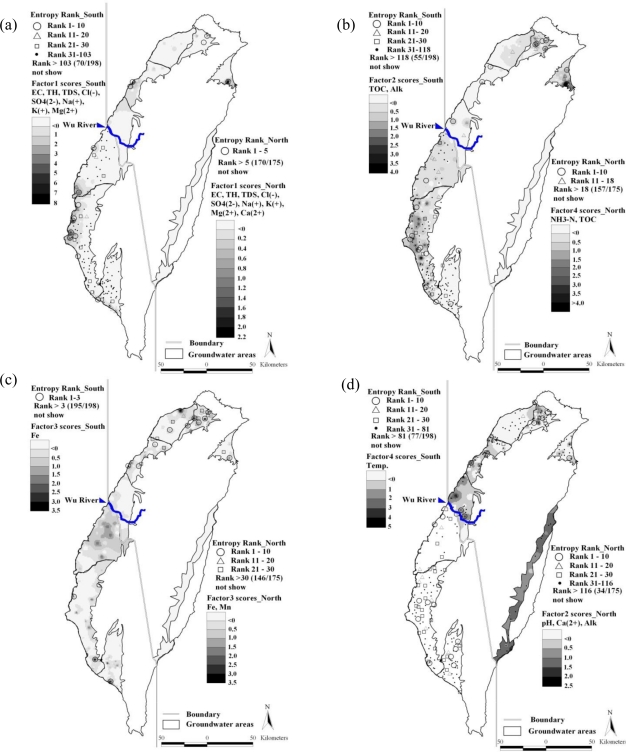
Overlay map of factor scores and information entropy values: **(a)** South, North-F1; **(b)** South-F2, North-F4; **(c)** South, North-F3; **(d)** South-F4, North-F2.

Figure 4(a) shows the overlaying map of the distribution of Factor 1 scores and the ranks of information entropy values for the northern and the southern areas. For the northern area, the ranks of information entropy values for only five of the monitoring wells vary noticeably. Unstable monitoring wells occupy 2.9% (5/175) of the total number of monitoring wells, located in Taipei City, Miaoli County, and Yilan County. The regions with high scores of Factor 1 conformed to the locations of these five unstable monitoring wells. In the southern area, the ranks of information entropy values showed that 103 monitoring wells had noticeable variation with 52.0% (103/198) of the monitoring wells being unstable. The overlay map reveals that the regions with high Factor 1 scores corresponded with monitoring well locations having the upper rankings of information entropy values. A higher concentration of the groundwater quality parameter contained in Factor 1 less stability. This is common to both the southern and the northern areas, but the problem is more serious in the southwestern coast of Taiwan because Factor 1 represents the extent of groundwater salinization. Thus, the information is useful to obtain the spatial distribution of groundwater salinization. Figure 4(a) reveals that the monitoring wells that have unstable groundwater quality located in potential areas of groundwater salinization are polluted.

Figure 4(b) shows the overlay map of the distributions of Factor 4 scores for the northern area and Factor 2 scores for the southern area, and the ranks of information entropy values. The ranks of information entropy values for the northern area exhibit that 18 monitoring wells have obvious variations with 10.3% (18/175) unstable monitoring wells aggregated in Taipei County and Ilan County. The regions with high Factor 4 scores conformed to monitoring well locations having upper rankings of information entropy values. Organic matter in these dense population areas pollutes the groundwater to interfere with groundwater quality stability. In the southern area, the ranks of information entropy values indicate that 118 monitoring wells have obvious variations with 59.6% (118/198) unstable monitoring wells spread extensively. The regions with high Factor 2 scores did not correspond with monitoring well locations having upper rankings of information entropy values, indicating that concentrations of TOC and Alk in the groundwater did not positively relate to groundwater quality stability. This is caused by the pollution source originating from animal husbandry, characterized by a wide range of a long-term, slow pollution.

Figure 4(c) shows the overlay map of the distribution of Factor 3 scores and the ranks of information entropy values for the northern and the southern areas. The ranks of information entropy values for the northern area show that 30 monitoring wells have obvious variations with 17.1% (30/175) unstable monitoring wells, located in Taipei City, Taipei County, Taoyuan County, Hsinchu County, and Miaoli County. The regions with high Factor 3 scores conformed to monitoring well locations having upper rankings of information entropy values. In the southern area, the ranks of information entropy values revealed that three monitoring wells have obvious variations with 1.5% (3/198) unstable monitoring wells located in Tainan County, Kaohsiung County, and Pingtung County. The overlay map revealed that the regions with high Factor 3 scores corresponded with monitoring well locations having upper rankings of information entropy values. Factor 3 is composed of iron and manganese ions in the northern area, while Factor 3 only contains iron ions in the southern area. These elements are natural components of soil and rocks commonly found in groundwater, indicating that natural variation is the main cause of unstable groundwater quality.

Figure 4(d) shows the overlay map of the distributions of Factor 2 scores for the northern area and Factor 4 scores for the southern area, and the ranks of information entropy values. The ranks of information entropy values for the northern area show that 116 monitoring wells having obvious variations with 66.3% (116/175) unstable monitoring wells. These monitoring wells are located in Taipei City, Taipei County, Hsinchu County, Miaoli County, and Taichung County. The regions with high Factor 2 scores conformed to monitoring well locations having upper rankings of information entropy values. In the southern area, the ranks of information entropy values reveals that 81 monitoring wells have obvious variations with 40.9% (81/198) unstable monitoring wells located extensively. The high Factor 4 scores are different from the upper ranking of information entropy values, indicating that natural causes influence groundwater temperature.

### 3.6. Serious Salinization Groundwater in Southwestern Taiwan

**Figure 5.**
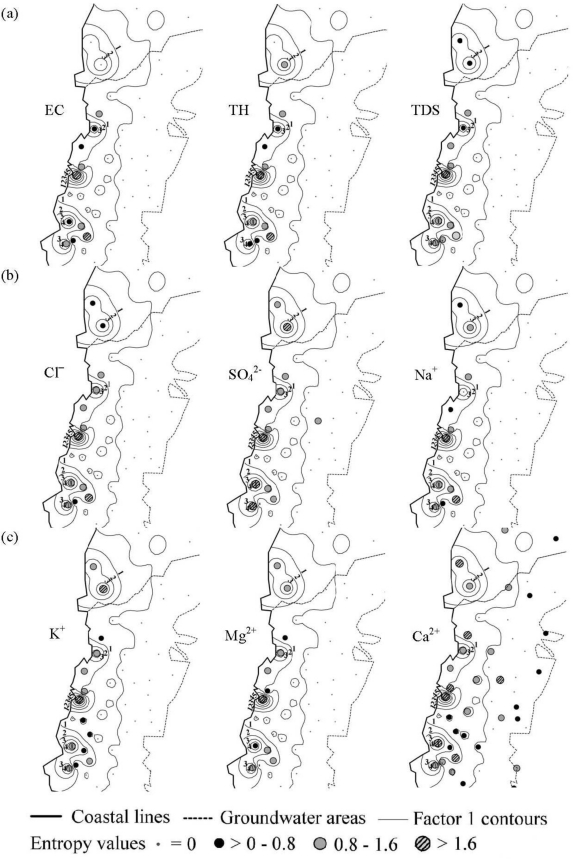

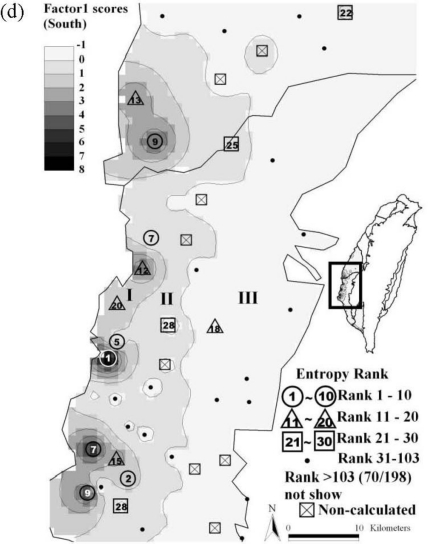
**(a)**∼**(c)** Distribution of the information entropy values for various hydrochemical parameters in common Factor 1; **(d)** ranks of information entropy values Factor 1 in Southwestern Taiwan.

To understand the extent of Factor 1 impact on groundwater quality in the southern area, the study area concentrated on monitoring wells located only in the coast area of Chianan Plain. Using the information entropy method to evaluate the stability of groundwater parameters in Factor 1 emphasized the correlation between factor scores and information entropy values as shown in Figures 5(a)–(c). Results of overlaying the contours of Factor 1 and the information entropy values of Factor 1 showed that all of the parameters except Ca^2+^ indicated that the monitoring wells with high information entropy values of parameters were located closely in the regions having high Factor 1 scores. This indicated that monitoring wells with high Factor 1 scores had relative poor groundwater quality stability. These monitoring wells aggregated in the coastal areas, so salinization obviously affected groundwater quality. Factor loading for Ca^2+^ was 0.729, lower than other parameters of Factor 1. However, Ca^2+^ fitted the criterion for selecting groundwater parameters, showing that Ca^2+^ is not a main control parameter in Factor 1. This finding could prove that additional analyses of groundwater quality uncertainty in this study assisted in understanding various groundwater parameters, and how the same pollution source affected those parameters.

Figure 5(d) reveals that the monitoring wells within the top 20 ranks of the information entropy values are mostly located in coastal areas. The Factor 1 scores for monitoring wells also gradually decreased from the coast to inland (the further the monitoring wells are from the coast, the smaller the Factor 1 scores). In contrast, the ranks of information entropy values gradually increased (the further the monitoring wells are from the coast, the lower the ranks of information entropy values). From left to right, the study area can be broadly divided into three zones based on Factor 1 scores. Table 6 shows the statistical ranks of Factor 1 information entropy values. The nine monitoring wells closest to the sea in zone I have high Factor 1 scores (Factor 1 scores 1–8) and high information entropy values. According to the ranks of information entropy values, five of the monitoring wells (56%) are within the top ten ranks and the other four wells are (44%) 11 to 20. In zone II, further away from the coastal area, 12 monitoring wells (58%) have smaller Factor 1 scores than zone I (Factor 1 scores 0–1) with six monitoring wells (50%) ranking 31–103; Only two wells (17%) rank within the top ten. In zone III, furthest away from the coastal area, the monitoring wells have the smallest Factor 1 scores (scores <0).

The basis of the ranks of these information entropy values are that 94% of the total 30 monitoring wells in zone III rank beyond 31, only 6 % rank between 21 to 30. This result indicates that the information entropy values for the monitoring wells in zone II are apparently smaller than in zone I. These observations infer that salinization seriously pollutes groundwater in Southwestern Taiwan and groundwater quality is very unstable, proving ongoing salinization. Monitoring wells located in zone II are also close to zone I, implying a continuous salinization factor seriously affecting groundwater quality, which is a potential area of groundwater quality deterioration. Therefore, these monitoring wells must monitor hydrochemical parameters of salinity regularly to prevent further groundwater quality deterioration. The unplanned development of aquaculture ponds and groundwater over-pumping in Taiwan coastal areas has caused an unbalanced supply/demand of fresh water in groundwater aquifers that has lead to inland seawater intrusion and an adverse influence of groundwater resources in the coastal area. The Taiwan Water Resources Agency (WRA) reported support the points as same as our study, the high water demand for aquaculture feeding consumed about 29% of all groundwater resources in Southwestern Taiwan. Serious over-pumping of groundwater has caused land subsidence and seawater intrusion to result in groundwater salinization.

### 3.7. Relations between the Factor Score and Information Entropy Value

**Figure 6.**
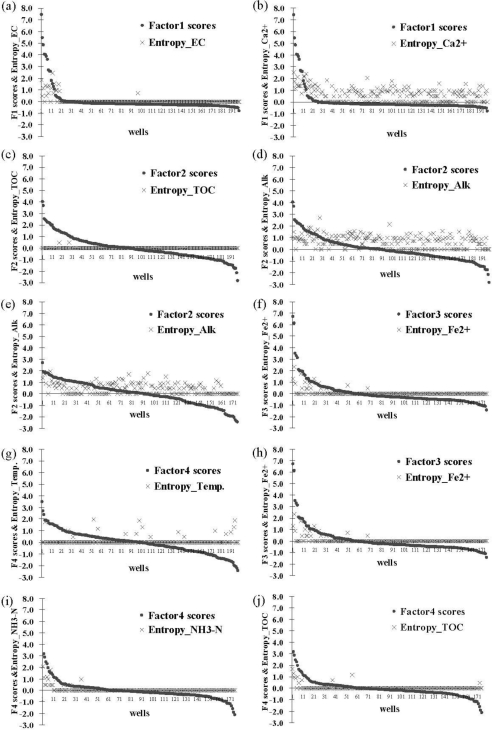
Relations between the factor score and information entropy value **(a)** South-EC, **(b)** South-Ca^2+^, **(c)** South-TOC, **(d)** South-Alk, **(e)** North-Alk, **(f)** South-Fe^2+^, **(g)** South-Temp., **(h)** North-Fe^2+^, **(i)** North-NH_3_-N, **(j)** North-TOC.

Figure 6 shows the relationship between factor scores and calculated information entropy values of the various hydrochemical parameters for each monitoring well in the northern area and the southern area. In the southern area, the relationship between Factor 1 scores and information entropy values are similar for Factor 1-contained hydrochemical parameters, except for Ca^2+^. Only the results for EC and Ca^2+^ are displayed in Figure 6(a) and Figure 6(b). The comparison of EC and Ca^2+^ reveals a specific relation between EC factor scores and information entropy values. In terms of Factor 1, the relations are similar for the northern and southern areas. The only difference is that in the southern area, there is no similar relation for the parameter Ca^2+^. Therefore, including Ca^2+^ in Factor 1 is not appropriate for the southern area. Figure 6(c) and Figure 6(d) show the results of TOC and Alk, which are parameters for Factor 2 in the southern area. The information entropy values of parameters TOC and Alk have no relationship with Factor 2 scores, indicating extensive groundwater pollution by organic matter. The regions where groundwater quality is unstable assist in finding the location of potential pollution areas. In the northern region, the Alk parameter in Factor 2 is similar to that in the southern area, and the Factor 2 score does not relate to the information entropy value shown in Figure 6(e). Both parameters Ca^2+^ and pH are also in Factor 2 in the northern area due to different sources. Hence, natural effects caused the instability of Alk in groundwater of the northern area.

In the southern area, Figure 6(f) and Figure 6(g) display the relationship between Factor 3 scores and information entropy values of parameter Fe^2+^, and Factor 4 scores and information entropy values of parameter temperature. Natural effects strongly influence these two factors, so that their factor scores and information entropy values do not correlate. Both parameters, Fe^2+^ and Mn^2+^ in the northern area have a similar relationship between factor scores and information entropy value, so that only the result for Fe^2+^ is shown in Figure 6(h). These findings demonstrate that the northern area is different from the southern area because Factor 3 scores highly correlate with information entropy values for parameters Fe^2+^ and Mn^2+^ in the northern area. Since Fe^2+^ and Mn^2+^ are natural elements, the groundwater that contains high concentrations of these elements has an unstable groundwater quality. This represents continuous natural variations, and observations show whether there are any other continuously changing natural phenomena in these regions. Figure 6(i) and Figure 6(j) show the results of NH_3_-N and TOC, which are parameters for Factor 4 in the northern area. Human activities highly influence Factor 4, as demonstrated by the weak relationship between Factor 4 scores and information entropy values of parameters NH_3_-N and TOC. Views of these regions revealed that cities with a high population density discharging improperly treated domestic wastewaters caused groundwater quality deterioration.

## 4. Conclusions

Overall, groundwater pollution in Taiwan is not serious. One hundred eight monitoring wells, accounting for 29.0% of total monitoring wells, possess the potential for groundwater salinization or have already been intruded by seawater, causing unstable groundwater quality. Groundwater over-pumping for aquaculture feeding in the southwestern coast of Taiwan causes ongoing seawater intrusion. Cluster analysis let us know that using the Wu River as a boundary, Taiwan can be divided into northern and southern areas with drastic differences in groundwater quality between the two areas. The southern area suffers from discharged agricultural and industrial wastewaters into the groundwater, causing groundwater pollution and higher EC concentration than the northern area. In the northern area, Taipei City, Taipei County, and Ilan County are densely populated areas. Discharges of domestic wastewater have already polluted the groundwater and interfered with groundwater quality stability.

Combining the geostatistical method and the information entropy theory to evaluate groundwater quality variations provides an effective tool for analyzing common factor uncertainty, and for conducting complete analyses of long-term monitoring data collected from a large-scale region. The results will facilitate subsequent researches on environmental remediation, pollution prevention, and investigating natural variations and implementing water management projects.

## Figures and Tables

**Table 1.** Summary statistics for hydrochemical data in Taiwan (unit: EC as *μ*S/cm, others as mg/L).

Groups		Temp.	pH	E.C.	TH	TDS	Cl ^−^	NH_4_-N	NO_3_-N	SO_4_^2−^	TOC	Fe^2+^	Mn^2+^

North	Min	18.2	3.6	70.0	5.5	44.5	N.D. ^a^	N.D. ^a^	N.D. ^a^	N.D. ^a^	0.2	N.D. ^a^	N.D. ^a^
(n = 1,796)	Median	25.2	6.4	498.0	176.0	318.0	19.4	0.1	0.8	55.8	2.1	0.8	0.8
	Max	30.5	9.9	27,200.0	4,890.0	24,800.0	11,300.0	55.2	24.8	1,330.0	91.8	140.0	12.9
	Mean	25.2	6.4	695.7	209.0	463.1	84.6	0.9	2.0	73.9	2.7	2.1	0.5
	S.D.	1.6	0.7	1,682.2	229.9	1,223.0	602.9	3.5	2.9	91.2	3.2	7.6	1.1

South	Min	22.0	5.5	168.0	32.4	120.0	N.D. ^a^	N.D. ^a^	N.D. ^a^	N.D. ^a^	0.1	N.D. ^a^	N.D. ^a^
(n = 2,013)	Median	27.2	7.0	899.0	378.0	591.0	34.4	0.1	0.1	100.0	1.5	0.18	0.4
	Max	32.4	10.5	82,300.0	14,400.0	64,200.0	31,900.0	37.4	65.4	5,420.0	9.7	16.6	8.0
	Mean	27.3	7.0	3,857.0	717.5	2,726.3	1,138.8	1.1	2.6	250.9	1.8	0.9	0.6
	S.D.	1.2	0.4	10,507.6	1,338.1	7,787.2	3,983.6	2.56	5.1	560.5	1.4	1.8	0.8

Total	Min	18.2	3.6	70.0	5.5	44.5	N.D. ^a^	N.D. ^a^	N.D. ^a^	N.D. ^a^	0.1	N.D. ^a^	N.D. ^a^
(n = 3,809)	Median	26.5	6.8	668.0	273.0	442.0	23.9	0.1	0.4	72.9	1.8	0.8	0.2
	Max	32.4	10.5	82,300.0	14,400.0	64,200.0	31,900.0	55.2	65.4	5,420.0	91.8	140.0	12.9
	Mean	26.3	6.7	2,366.4	477.7	1,659.2	641.7	1.0	2.3	167.5	2.3	1.4	0.5
	S.D.	1.7	0.6	7,884.2	1,017.6	5,832.9	2972.0	3.0	4.2	421.6	2.4	5.4	0.9

	MDL	–	–	–	5	–	1.6	0.02	0.01	1.0	0.05	0.005	0.005

	< N.D.(n) ^a^	0	0	0	0	0	17	853	444	32	1	310	589

Groups		Na^+^	K^+^	Ca^2+^	Mg^2+^	Alk	As ^b^	Cd ^b^	Cr ^b^	Cu ^b^	Pb ^b^	Zn ^b^	

North	Min	2.6	0.1	0.57	N.D.	N.D. ^a^	N.D. ^a^	N.D. ^a^	N.D. ^a^	N.D. ^a^	N.D. ^a^	N.D. ^a^	

(n = 1,796)	Median	23.6	2.8	45.70	13.3	141.5	N.D. ^a^	N.D. ^a^	N.D. ^a^	N.D. ^a^	N.D. ^a^	0.020	

	Max	7,380.0	257.0	308.00	742.0	744.0	0.185	0.018	0.022	0.211	0.082	7.980	

	Mean	65.6	5.1	52.77	18.9	157.3	0.003	0.001	0.002	0.003	0.003	0.050	

	S.D.	359.3	13.3	38.53	39.0	104.6	0.010	0.001	0.001	0.007	0.004	0.207	

South	Min	1.9	0.1	4.29	0.4	11.1	N.D. ^a^	N.D. ^a^	N.D. ^a^	N.D. ^a^	N.D. ^a^	N.D. ^a^	

(n = 2,013)	Median	39.1	4.5	106.00	24.6	299.0	0.002	N.D. ^a^	N.D. ^a^	N.D. ^a^	N.D. ^a^	0.003	

	Max	15,200.0	1,040.0	630.00	3,070.0	1,030.0	0.191	0.009	0.032	0.484	0.104	0.334	

	Mean	618.5	30.0	125.80	93.0	308.4	0.009	0.001	0.002	0.002	0.003	0.007	

	S.D.	2,054.4	91.1	84.00	268.6	137.1	0.019	0.001	0.002	0.011	0.005	0.015	

Total	Min	1.9	0.1	0.57	N.D. ^a^	N.D. ^a^	N.D. ^a^	N.D. ^a^	N.D. ^a^	N.D. ^a^	N.D. ^a^	N.D. ^a^	

(n = 3,809)	Median	28.2	3.4	78.40	17.6	219.0	0.001	N.D. ^a^	N.D. ^a^	N.D. ^a^	N.D. ^a^	0.007	

	Max	15,200.0	1,040.0	630.00	3,070.0	1,030.0	0.191	0.018	0.032	0.484	0.104	7.980	

	Mean	357.8	18.3	91.37	58.0	237.1	0.006	0.001	0.002	0.003	0.003	0.027	

	S.D.	1,538.5	68.0	75.87	200.5	144.2	0.016	0.001	0.002	0.010	0.005	0.144	

	MDL	0.1	0.1	0.1	0.1	0.1	0.0005	0.001	0.005	0.005	0.005	0.005	

	< N.D.(n) ^a^	0	0	0	3	5	1,553	3,457	3,539	3,336	3,429	838	

a N.D. represents values are lower than method detection limit (MDL);

b Values for constituents lower than MDL were replaced with MDL/2.

**Table 2.** The correlation coefficient matrix for hydrochemical parameters.

	**Temp.**	**pH**	**EC**	**TH**	**TDS**	**Cl^−^**	**NH_4_-N**	**NO_3_-N**	**SO_4_^2^**	**TOC**	**Fe^2+^**	**Mn^2+^**	**Na^+^**	**K^+^**	**Ca^2+^**	**Mg^2+^**	**Alk**	**As**	**Cd**	**Cr**	**Cu**	**Pb**	**Zn**
**Temp.**	1.000 *																						
**pH**	0.364 *	1.000																					
**EC**	0.187 *	0.175 *	1.000 *																				
**TH**	0.239 *	0.205 *	0.970 *	1.000																			
**TDS**	0.181 *	0.166 *	0.998 *	0.979 *	1.000																		
**Cl^−^**	0.165 *	0.152 *	0.998 *	0.970 *	0.998 *	1.000																	
**NH_4_-N**	0.010	0.119 *	0.256 *	0.217 *	0.238 *	0.241 *	1.000																
**NO_3_-N**	0.042	−0.066	−0.120 *	−0.097 *	−0.114 *	−0.117 *	−0.186 *	1.000															
**SO_4_^2−^**	0.212 *	0.150 *	0.964 *	0.972 *	0.969 *	0.962 *	0.154 *	−0.093	1.000														
**TOC**	−0.229 *	−0.073	−0.050	−0.064	−0.058	−0.060	0.567 *	−0.230 *	−0.083	1.000													
**Fe^2+^**	−0.071	−0.193 *	0.071	0.075	0.072	0.070	0.288 *	−0.173 *	0.083	0.299 *	1.000												
**Mn^2+^**	0.144 *	−0.095	0.243 *	0.257 *	0.240 *	0.231 *	0.106 *	−0.169 *	0.299 *	0.169 *	0.465 *	1.000											
**Na^+^**	0.164 *	0.159 *	0.997 *	0.960 *	0.996 *	0.996 *	0.251 *	−0.120 *	0.955 *	−0.051	0.066	0.223 *	1.000										
**K^+^**	0.177 *	0.162 *	0.966 *	0.972 *	0.973 *	0.971 *	0.221 *	−0.115 *	0.950 *	−0.041	0.063	0.200 *	0.960 *	1.000									
**Ca^2+^**	0.434 *	0.414 *	0.670 *	0.752 *	0.672 *	0.646 *	0.164 *	−0.027	0.714 *	−0.087	0.010	0.293 *	0.645 *	0.610 *	1.000								
**Mg^2+^**	0.179 *	0.145 *	0.965 *	0.988 *	0.977 *	0.971 *	0.218 *	−0.103 *	0.961 *	−0.051	0.088	0.228 *	0.960 *	0.986 *	0.647 *	1.000							
**Alk**	0.462 *	0.553 *	0.189 *	0.241 *	0.172 *	0.144 *	0.277 *	−0.137 *	0.201 *	0.240 *	−0.038	0.172 *	0.163 *	0.181 *	0.521 *	0.165 *	1.000						
**As**	0.112 *	0.247 *	0.176 *	0.180 *	0.168 *	0.164 *	0.203 *	−0.224 *	0.147 *	0.205 *	0.166 *	0.061	0.170 *	0.176 *	0.192 *	0.165 *	0.286 *	1.000					
**Cd**	−0.102 *	−0.114 *	−0.011	−0.021	−0.013	−0.011	0.143	−0.065	−0.017	0.157 *	0.528 *	0.202 *	−0.011	−0.011	−0.064	−0.009	−0.044	0.261 *	1.000				
**Cr**	0.002	0.005	−0.022	−0.025	−0.021	−0.019	−0.032	0.047	−0.029	−0.041	−0.028	−0.050	−0.020 *	−0.023	−0.050	−0.018	−0.050	−0.036	−0.013	1.000			
**Cu**	−0.097 *	−0.071	−0.029	−0.034	−0.028	−0.031	−0.005	0.023	−0.027	0.102 *	−0.011	0.029	−0.027	−0.023	−0.051	−0.029	0.032	−0.051	0.185 *	−0.017	1.000		
**Pb**	0.011	0.016	−0.012	−0.003	−0.011	−0.014	−0.011	0.204 *	−0.001	0.044	0.004	−0.009	−0.014	−0.009	0.027	−0.007	0.030	−0.005	0.085	−0.007	0.256 *	1.000	
**Zn**	−0.213 *	−0.432 *	−0.077	−0.095	−0.073	−0.068	−0.070	−0.096	−0.049	0.159 *	0.280 *	0.137 *	−0.070	−0.067	−0.209 *	−0.065	−0.270 *	−0.099 *	0.311 *	−0.023	0.143 *	−0.012	1.000

a Significant correlations (>0.70) in bold.

* p-value < 0.05.

**Table 3.** The rotated common factors for loadings, the percentage of variance and the total cumulative percentage of variance in the southern and northern areas.

**Parameter**	**North ^a^**				**South ^a^**			
	**Factor 1**	**Factor 2**	**Factor 3**	**Factor 4**	**Factor 1**	**Factor 2**	**Factor 3**	**Factor 4**
Temp.	0.079	0.200	0.168	−0.277	0.057	0.146	−0.201	**0.738**
pH	0.065	**0.814**	−0.180	0.017	0.172	0.512	−0.339	−0.486
EC	**0.985**	0.120	0.043	0.089	**0.987**	0.070	0.077	0.024
TH	**0.887**	0.423	0.110	−0.032	**0.981**	0.043	0.120	0.072
TDS	**0.991**	0.108	0.030	0.033	**0.991**	0.050	0.084	0.029
Cl^−^	**0.991**	0.027	−0.012	0.068	**0.990**	0.042	0.077	0.018
NH_4_^−^N	0.191	0.124	0.180	**0.864**	0.351	0.505	0.262	−0.160
NO_3_^−^N	−0.033	−0.177	−0.437	−0.158	−0.060	−0.430	−0.574	0.113
SO_4_^2−^	**0.669**	0.207	0.359	−0.408	**0.974**	0.032	0.088	0.110
TOC	0.080	0.257	0.369	**0.777**	−0.103	**0.813**	0.093	0.172
Fe^2+^	0.078	−0.178	**0.810**	0.193	0.239	−0.072	**0.809**	0.087
Mn^2+^	0.009	−0.023	**0.892**	−0.075	0.294	0.105	0.319	0.597
Na^+^	**0.990**	0.039	0.008	0.089	**0.984**	0.062	0.066	0.003
K^+^	**0.962**	0.104	−0.023	0.028	**0.971**	0.073	0.065	0.014
Ca^2+^	0.332	**0.849**	0.069	−0.071	**0.729**	0.096	0.239	0.239
Mg^2^	**0.965**	0.128	0.139	0.076	**0.977**	0.028	0.095	0.036
Alk	0.100	**0.852**	0.190	0.373	0.046	**0.800**	−0.044	0.168
Eigenvalue	7.19	2.57	2.08	1.84	8.56	2.07	1.43	1.32
Total variance (%)	42.28	15.13	12.21	10.80	50.34	12.19	8.44	7.76
Cumulative variance (%)	42.28	57.41	69.62	80.42^b^	50.34	62.53	70.97	78.73 ^b^

a The loadings whose absolute value is more than 0.625 of the total variance were in bold;

b Total cumulative variances.

**Table 4.** Variography results for factor scores.

**Region**	**Common factor**	**Model type**	**C_0_**	**C_0_ + C**	**Range**	**R^2^**	**RSS**
North	F1	Linear	0.750	0.750	243,217	0.627	0.975
	F2	Exponential	0.384	1.011	24,300	0.753	0.050
	F3	Gaussian	0.001	1.132	1,663	0.527	0.872
	F4	Gaussian	0.506	3.022	201,784	0.689	0.854
South	F1	Gaussian	0.497	3.004	152,767	0.883	0.432
	F2	Gaussian	0.510	1.344	50,749	0.957	0.057
	F3	Exponential	0.146	1.048	4,920	0.699	0.025
	F4	Exponential	0.658	1.547	165,900	0.872	0.044

RSS: Residual sum of square. C_0_: Nugget. C_0_ + C: Sill.

**Table 5.** Statistics on the ranks of information entropy values for common factors.

**ID**	**Sample name**	**pH entropy value(rank)**	**Ca^2+^ entropy value(rank)**	**Alk. entropy value(rank)**	**Sum of ranks**	**Factor2 rank**
1	Ab026	1.000 (4)	1.571 (2)	1.685 (5)	11	1
2	Dg147	1.971 (1)	0.971 (12)	0.971 (22)	35	2
3	Aa009	1.485 (3)	0.722 (31)	1.771 (4)	38	3
4	Dj166	1.000 (4)	0.722 (31)	1.000 (16)	51	4
:	:	:	:	:	:	:
:	:	:	:	:	:	:
174	Hs393	0.000 (89)	0.000 (70)	0.000 (99)	258	146
175	Cf130	0.000 (89)	0.000 (70)	0.000 (99)	258	146

Factor 2 for the northern area is taken as an example.

**Table 6.** Statistics on the ranks of information entropy values for Factor 1 on the southwestern coast of Taiwan.

**Factor1 entropy rank interval**	**Zone I (location F1 score 1–8)**	**Zone II (location F1 score 0–1)**	**Zone III (location F1 score <0)**
Rank 1–10	5/9 (56%)	2/12 (17%)	0/30 (0%)
Rank 11–20	4/9 (44%)	0/12 (0%)	1/30 (3%)
Rank 21–30	0/9 (0%)	3/12 (25%)	1/30 (3%)
Rank 31–103	0/9 (0%)	6/12 (50%)	14/30 (47%)
Rank > 103	0/9 (0%)	1/12 (8%)	14/30 (47%)

Based on Figure 5(d).
